# Endovascular Management of Iliac Hematoma Associated with May–Thurner Syndrome Using Mechanical Thrombectomy and Bare-Metal Stenting: A Case Report

**DOI:** 10.3390/jcm15031263

**Published:** 2026-02-05

**Authors:** HyeRee Cho, YooJin Nam, Pa Hong, YangWon Kim

**Affiliations:** Department of Radiology, Samsung Changwon Hospital, Sungkyunkwan University School of Medicine, Changwon 51353, Republic of Korea

**Keywords:** May–Thurner syndrome, deep vein thrombosis, iliac hematoma, mechanical thrombectomy, bare-metal stent

## Abstract

**Background/Objectives:** May–Thurner syndrome (MTS) is a common cause of iliofemoral deep vein thrombosis (DVT). Venous bleeding associated with MTS is extremely rare and has been reported mainly as spontaneous iliac vein rupture (SIVR) with retroperitoneal or iliac hematoma. Additionally, standardized treatment strategies have not yet been established. Herein, we report a case of an iliac hematoma associated with MTS that was successfully treated with endovascular mechanical thrombectomy and bare-metal stenting. **Case Presentation:** A 69-year-old man presented with acute swelling and pain in the left lower extremity. Computed tomography angiography demonstrated extensive iliofemoral DVT and an iliac hematoma adjacent to the left common iliac vessels, without definite evidence of iliac vein rupture. Initial conservative management with anticoagulation therapy was selected as the patient was hemodynamically stable and showed no active bleeding. However, follow-up imaging one week later revealed persistent DVT with interval enlargement of the hematoma. Pelvic arteriography excluded an arterial bleeding source. Endovascular treatment was performed, including mechanical thrombectomy using the AngioJet system and bare-metal stent placement to restore venous outflow. Follow-up imaging revealed complete thrombus resolution, hematoma regression, and sustained stent patency. **Conclusions:** Iliac hematomas associated with MTS may occur without definite radiological evidence of iliac vein rupture. In selected hemodynamically stable patients, an individualized endovascular strategy focused on venous outflow restoration using the AngioJet system and bare metal stents may be a feasible treatment option.

## 1. Introduction

May–Thurner syndrome (MTS) is a common cause of iliofemoral deep vein thrombosis (DVT), resulting from chronic compression of the left common iliac vein (CIV) by the overlying right common iliac artery (CIA). Although extremely rare, MTS can be complicated by venous bleeding, which has been reported as spontaneous iliac vein rupture (SIVR), often presenting as a retroperitoneal or iliac hematoma. The pathogenesis of SIVR or iliac hematoma associated with MTS is not fully understood. However, multiple factors, including DVT, venous wall inflammation, venous hypertension, and decreased vessel wall elasticity, have been proposed as contributing factors [1].

To date, fewer than 71 cases of SIVR have been reported in the literature; most were treated surgically, whereas only a small number were managed conservatively or with endovascular treatment [1,2,3,4,5,6,7,8]. Due to its rarity, clear treatment guidelines have not yet been established, making clinical management challenging. Herein, we report a case of iliac hematoma associated with MTS that was successfully treated with endovascular mechanical thrombectomy and bare-metal stenting.

## 2. Case Report

A 69-year-old man presented in April 2025 with acute swelling and pain in the left lower extremity without a history of recent trauma. The patient had a history of nasal cavity melanoma treated with surgical resection 16 years prior as well as dementia. He was diagnosed with vasovagal syncope, managed conservatively, and was not receiving any vasoactive agents, anticoagulants, or antiplatelet therapy at presentation. He was hemodynamically stable. Physical examination revealed diffuse pitting edema and tenderness without skin discoloration. Peripheral pulses were preserved with no cardiopulmonary symptoms or prior thromboembolic events.

Initial laboratory results showed a hemoglobin level of 11.0 g/dL (reference range, 12.7–17.3 g/dL) and a markedly elevated D-dimer level of 10,881 ng/mL (reference range, <250 ng/mL). His international normalized ratio was slightly elevated at 1.21 (reference range, 0.8–1.2). Other coagulation parameters, including platelet count and activated partial thromboplastin time, were within normal limits.

**Figure 1:**
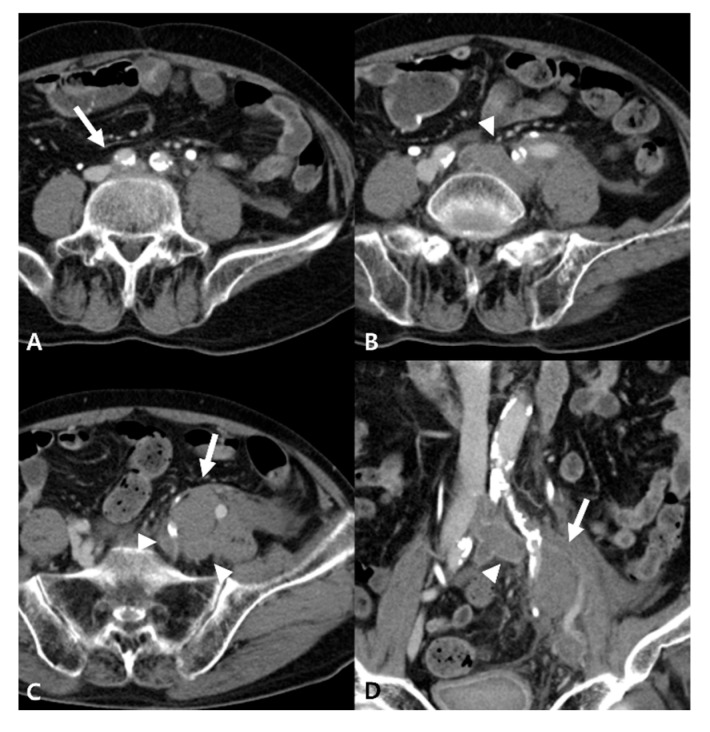
Initial contrast-enhanced CT angiography. (**A**) Delayed-phase CT demonstrates compression of the left CIV by the overlying right CIA (arrow). (**B**–**D**) Extensive DVT involving the left CIV, internal iliac vein, and EIV (arrowheads), with an adjacent non-enhancing low-attenuation lesion consistent with an iliac hematoma (arrow).

Initial computed tomography (CT) angiography revealed compression of the left common iliac vein by the overlying right CIA, resulting in approximately 73.33% luminal stenosis. Extensive iliofemoral DVT involving the left CIV, external iliac vein (EIV), and common femoral vein (CFV), and extension to the popliteal vein was also noted. A non-enhancing, low-attenuation lesion (3.5 × 2.5 cm) was identified adjacent to the proximal left iliac vessels and was suspected to be a hematoma associated with iliac vein rupture (Figure 1). In addition, pulmonary thromboembolism was observed in the subsegmental pulmonary artery of the right lower lobe. Based on the imaging findings, the patient underwent prompt placement of an inferior vena cava (IVC) filter after admission to prevent thrombus migration to the pulmonary arteries.

The concomitant presence of DVT and a suspected hematoma posed a therapeutic dilemma. The patient was hemodynamically stable, his hemoglobin level was within the normal range, and no evidence of active bleeding or definite iliac vein rupture was identified on contrast-enhanced CT. Thus, we initially opted for conservative management with low-molecular-weight heparin (Enoxaparin, 80 mg twice daily) and close observation. His D-dimer level markedly decreased to 1032 ng/mL within 4 days of anticoagulation; however, one week later, it increased to 2047 ng/mL, accompanied by a slight decrease in the hemoglobin level to 9.6 g/dL. Additionally, the patient’s symptoms persisted.

**Figure 2:**
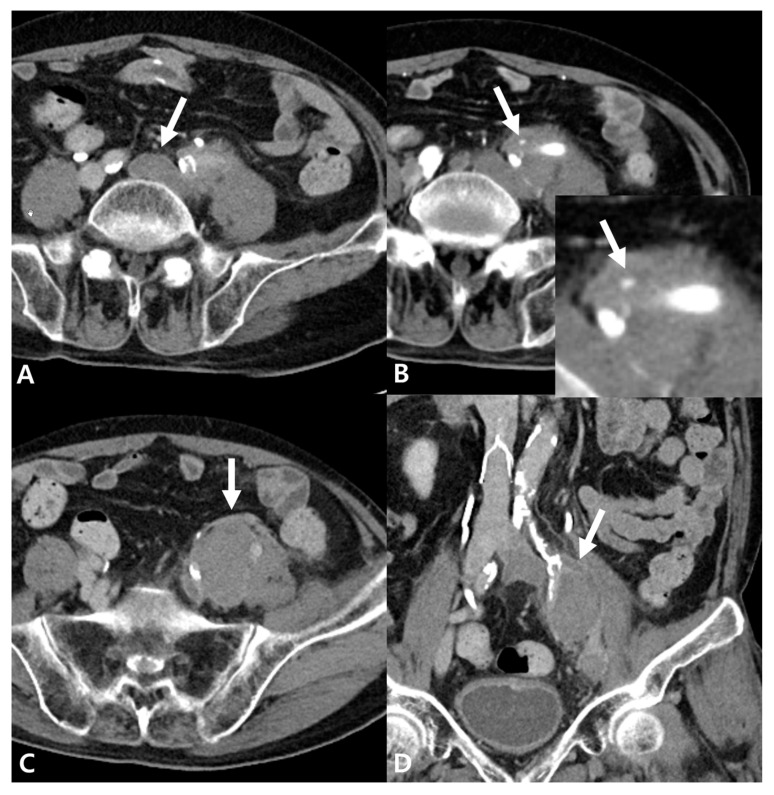
Follow-up contrast-enhanced CT performed one week after admission. (**A**) There is no significant interval change in the previously identified DVT (arrow) involving the left CIV to CFV. (**B**) Arterial phase CT demonstrates a small enhancing focus (arrow) within the iliac hematoma, raising concerns for a possible arterial bleeding source, such as a pseudoaneurysm. (**C**,**D**) There is an interval increase in the size of the suspected iliac hematoma (arrows) adjacent to the left iliac vessels.

Given these laboratory changes and persistent symptoms, a follow-up CT angiography was performed, which demonstrated persistent extensive DVT involving the left CIV, EIV, and CFV, with only partial improvement of the femoral and popliteal vein thrombosis despite anticoagulation. The previously identified iliac hematoma had increased in size to 4.5 × 3.2 cm, with an enhancing focus noted within the hematoma on the arterial phase of contrast-enhanced CT (Figure 2).

**Figure 3:**
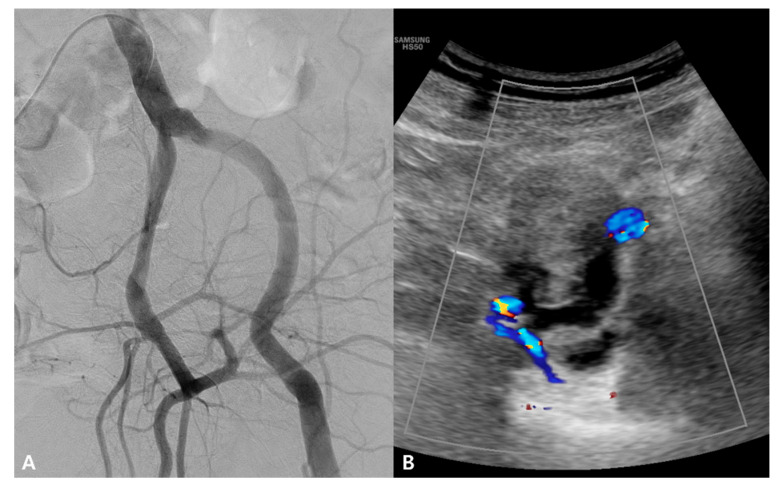
Pelvic arteriography and Doppler ultrasonography. (**A**) Selective arteriography of the left CIA, internal iliac artery, and EIA obtained in a 22° RAO projection demonstrates no definite contrast extravasation or pseudoaneurysm. (**B**) Concomitant Doppler ultrasonography shows a heteroechoic lesion between the left EIA and internal iliac artery, consistent with a hematoma, without evidence of active bleeding (colors indicate the left external and internal iliac arteries).

Although the hematoma was suspected to be secondary to an iliac vein injury associated with MTS, pelvic arteriography was performed to exclude potential sources of arterial bleeding, such as a small pseudoaneurysm. However, no definite contrast extravasation or pseudoaneurysm was observed. Doppler ultrasonography performed concurrently with pelvic arteriography in an interventional suite revealed a hematoma without any evidence of active bleeding (Figure 3).

**Figure 4:**
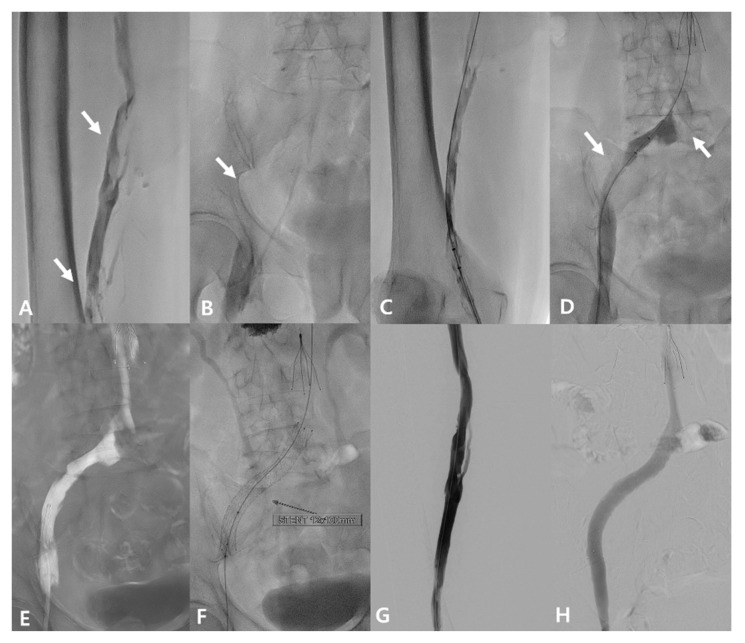
Endovascular procedure with mechanical thrombectomy and bare-metal stenting. (**A**) Initial venography demonstrates multifocal partial thrombosis involving the left femoral and popliteal veins (arrows). (**B**) Venography shows extensive DVT (arrow) involving the left CIV to CFV. (**C**,**D**) Rheolytic thrombectomy using the AngioJet system was performed from the left femoral vein to CIV. Residual thrombus is observed (arrow), with no definite contrast extravasation. (**E**) Limited aspiration thrombectomy using an 8-Fr guiding catheter was performed for residual thrombus. (**F**) A 12-mm × 100-mm bare-metal stent with strong radial force was deployed across the stenotic segment from the left CIV to the EIV to cover potential venous wall injury. (**G**,**H**) Final venography demonstrates restored antegrade venous flow from the left femoral vein to the IVC without residual filling defects or contrast extravasation.

Based on these imaging findings and persistent symptoms, an endovascular intervention was planned. The patient was placed in the prone position. Venous access was obtained through the left popliteal vein using a 9-Fr sheath. Initial venography demonstrated extensive DVT involving the left CIV to CFV with partial thrombosis of the superficial femoral vein (SFV) and popliteal veins, which limited an accurate evaluation of a possible iliac vein rupture (Figure 4A,B).

A 0.035-inch, 260-cm hydrophilic guidewire (Terumo, Tokyo, Japan) was carefully negotiated with a 4-fr angiographic catheter (Berenstein; Merit Medical, South Jordan, UT, USA) through the narrowed left CIV. Rheolytic thrombectomy using the AngioJet system (Boston Scientific, Marlborough, MA, USA) was performed from the left femoral vein to the CIV without the use of a recombinant tissue plasminogen activator because of concerns regarding the bleeding risk associated with the concomitant hematoma. Post-thrombectomy venography revealed no evidence of contrast extravasation (Figure 4C,D).

Subsequently, limited aspiration thrombectomy was performed for the residual thrombus using an 8-fr guiding catheter (Vista Brite Tip guide catheter; Cordis, Miami Lakes, FL, USA). Pre-dilatation of the left CIV was then performed using a 12-mm balloon, followed by deployment of a 12-mm × 100-mm self-expanding venous stent (Venovo; BD, Tempe, AZ, USA) across the stenotic segment, extending to cover any potential leakage point (Figure 4E,F). Final venography showed restored antegrade flow through the left femoral vein to the stented left iliac vein segment without residual filling defects or contrast extravasation (Figure 4G,H). There were no procedure-related complications during or after the intervention.

**Figure 5:**
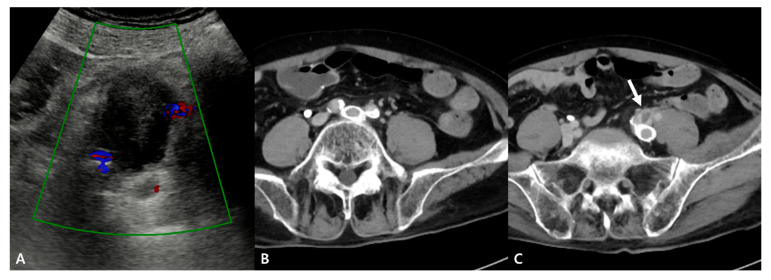
Follow-up ultrasonography and CT. (**A**) Six days after the intervention, follow-up Doppler ultrasonography demonstrates a resolving hematoma (colors indicate the left external and internal iliac arteries). (**B**,**C**) At the 3-month follow-up visit, delayed-phase CT demonstrates patency of the left CIV stent, with resolution of DVT and a markedly decreased size of the resolving hematoma adjacent to the iliac vessels (arrow).

Six days after the intervention, the IVC filter was removed. At the time of filter removal, follow-up Doppler ultrasonography demonstrated no interval change in size, but decreased echogenicity of the lesion, consistent with a resolving hematoma (Figure 5A). The patient was discharged two days later. After discharge, anticoagulation therapy with rivaroxaban was prescribed at 15 mg twice daily for 2 weeks, followed by 20 mg once daily for 3 months.

At the 3-month follow-up visit, the swelling of the left leg had completely improved, and contrast-enhanced CT demonstrated complete resolution of DVT with sustained stent patency, resolved pulmonary thromboembolism, and a marked decrease in the size of the resolving hematoma adjacent to the left iliac vessels (Figure 5B,C). The patient has remained asymptomatic for 7 months and his D-dimer level has normalized.

## 3. Discussion

MTS is a well-established cause of iliofemoral venous outflow obstruction and often presents as extensive thrombosis when left untreated. However, venous bleeding associated with MTS is rare and is usually reported as spontaneous iliac vein rupture presenting with a retroperitoneal or iliac hematoma [1,2].

Management options for SIVR include surgical, endovascular, and conservative approaches. In a literature review of 68 reported cases, 42 patients (61.8%) underwent open iliac vein repair, 12 (17.6%) were treated with endovascular approaches, eight (11.8%) underwent ligation or Palma–Dale bypass, and six (8.8%) were managed conservatively [1]. Several reports have suggested that conservative management with anticoagulation therapy is a viable and safe alternative treatment option for hemodynamically stable patients without evidence of active bleeding [3,4,5]. In the present case, the patient was hemodynamically stable and presented with an iliac hematoma without a definite focus of iliac vein rupture on the initial contrast-enhanced CT scan. Considering these findings, anticoagulation therapy was initiated as first-line treatment, rather than immediate surgical or endovascular intervention. However, the patient’s symptoms did not improve, and a follow-up CT performed one week later demonstrated persistent DVT with an interval increase in the size of the iliac hematoma.

Given the presence of an enhancing focus within the hematoma on arterial-phase CT, pelvic arteriography was performed to exclude potential arterial bleeding sources, which revealed no definite contrast extravasation. Based on these findings, the enhancing focus was considered more likely to represent enhancement of a small adjacent artery compressed or displaced by the mass effect of the hematoma, rather than a clinically significant arterial injury. Following the exclusion of arterial bleeding, the iliac hematoma was presumed to be related to occult or microscopic venous injury in the setting of venous hypertension and DVT associated with MTS.

Although anticoagulation may have contributed to enlargement of the iliac hematoma, persistent symptoms, ongoing extensive DVT on follow-up CT and re-elevation of D-dimer levels suggested that continuation of anticoagulation alone was unlikely to be sufficient. Accordingly, after the failure of conservative management with anticoagulation and consideration of these clinical and imaging findings, endovascular treatment was selected over an immediate surgical approach. A similar staged management strategy was reported by Nishimoto et al. [6]; specifically, a case of SIVR associated with MTS was initially managed conservatively and subsequently treated with elective endovascular intervention after the failure of conservative therapy.

Endovascular treatment options for MTS include catheter-directed thrombolysis (CDT), mechanical thrombectomy, angioplasty, and stent placement. CDT has been shown to effectively reduce thrombus burden and significantly decrease the risk of post-thrombotic syndrome [9]. However, CDT is contraindicated in patients with a bleeding diathesis.

Several studies have reported that pharmacomechanical thrombectomy using the AngioJet rheolytic thrombectomy system, either alone or in combination with CDT, is a safe and effective treatment for MTS [10,11,12]. Hui Zheng et al. [8] described two cases of SIVR–associated MTS that were managed using the AngioJet system followed by covered stent placement. Although the AngioJet generates high-velocity jets that may theoretically impose stress on the venous wall, extensive mechanical manipulation with large-bore aspiration devices may pose a greater risk of direct venous injury in certain settings. In this case, the AngioJet system was used in the thrombectomy-only mode primarily for thrombus debulking, followed by limited aspiration thrombectomy of the residual thrombus to minimize the overall mechanical manipulation of the venous wall.

After successful debulking of the thrombus burden, no definite rupture point was identified on venography. Unlike previous reports that focused on sealing iliac vein ruptures using covered stents [6,7,8], our strategy aimed to restore venous outflow and reduce venous hypertension, which is considered the primary mechanism of ongoing venous bleeding. Although covered stents may be appropriate in cases of definite venous rupture, their routine use in the absence of clear extravasation remains debatable, particularly in cases like ours. To the best of our knowledge, no direct comparative studies between covered- and bare-metal stents, specifically for the treatment of MTS, have been reported. In the present case, longer covered stents might have been required to cover potential venous wall injury sites, which could have unnecessarily occluded the internal iliac vein and other venous collateral pathways. In addition, covered stents are more expensive than bare-metal stents in Korean clinical practice. Considering these factors, a bare-metal stent with a strong radial force (Venovo stent) was selected, rather than a covered stent, and deployed across the stenotic segment of the left CIV, extending into the external iliac vein. To ensure adequate coverage of any potential venous wall injury, a 12-mm × 100-mm stent was used.

Currently, the optimal duration of anticoagulation therapy in patients with SIVR or iliac hematoma is not clearly defined. In the present case, the patient was treated with rivaroxaban for three months in accordance with the established recommendations for DVT.

This case report has several limitations. Evidence supporting the safety and efficacy of the AngioJet system in SIVR or iliac hematoma remains limited. Bare metal stents do not provide immediate sealing of venous wall defects and may not be appropriate for patients with confirmed venous rupture; their long-term efficacy in this setting has not been well established.

Although no standardized treatment strategy has been established, optimal management should be individualized based on each patient’s clinical condition and imaging findings, with careful selection of the most appropriate therapeutic approach for the given scenario.

## 4. Conclusions

The present case demonstrates that an iliac hematoma associated with MTS can occur without definite CT or angiographic evidence of iliac vein rupture. An individualized endovascular approach focusing on restoring venous outflow may be effective in select hemodynamically stable patients with concomitant DVT and hematoma. Mechanical thrombectomy without thrombolytic agents followed by bare metal stent placement may represent a reasonable treatment option when a clear venous rupture is not demonstrated.

## Data Availability

The original contributions presented in this study are included in the article. For further inquiries, please contact the corresponding author.

## Abbreviations

The following abbreviations are used in this manuscript:

CDT	Catheter-directed thrombolysis
CFV	Common femoral vein
CIA	Common iliac artery
CIV	Common iliac vein
DVT	Deep vein thrombosis
EIV	External iliac vein
IVC	Inferior vena cava
MTS	May–Thurner syndrome
SIVR	Spontaneous iliac vein rupture
